# Pre-Hospital Emergency Medical Services in Developing Countries: A Case Study about EMS Response Time in Yazd, Iran

**Published:** 2011-10-01

**Authors:** M A Bahrami, A Maleki, M Ranjbar Ezzatabadi, R Askari, G H Ahmadi Tehrani

**Affiliations:** 1 Department of Healthcare Management, Shahid Sadoughi University of Medical Sciences, Yazd, Iran; 2 Department of Healthcare Management, Islamic Azad University, Science and Research Branch, Tehran, Iran; 3 Department of Health Management and Economics, School of Public Health, Tehran University of Medical Sciences, Tehran, Iran

**Keywords:** Pre-hospital, Emergency medical services, Response time, Iran

## Abstract

**Background:**

Prehospital emergency medical services, a subsystem of Emergency Medical Services (EMS),provides early care to critically ill and injured patients in the field. Time is an important factor in pre-hospital care and the length of time it takes to reach care, has a significant impact on patient outcome. This study aims to calculate the response time in EMS stations of Yazd, Iran.

**Methods:**

During 2008-2009, 11961 run report forms from all 7 EMS stations in Yazd that had been submitted to central station were surveyed. By using Excell statistical software and descriptive statistics (percent, cumulative distribution and standard deviation), we calculated fractile response times for Yazd EMS.

**Results:**

81.15 percent of response times were within 8 minutes as a referenced standard for EMS.

**Conclusion:**

The majority of call services in Yazd EMS have been responded in a suitable time interval comparing with reference standards and country's approved EMS response time goal, but there are still rooms for improvement.

## Introduction

Injuries continue to grow as a cause of death and disability globally[1] and account for about 12% of the world's burden of disease.[2] In the 2003 World Health Organization Report, the WHO drew attention to the increasing burden of non-communicable and chronic diseases, trauma and depression. This shifting burden of disease has led the WHO to call for ''rapid and sustainable expansion of emergency treatments."[3]

In the most communities, this emergency treatment is provided by Emergency Medical Services (EMS).[4] EMS is the umbrella term for a continuum of health services including pre-hospital medical services, emergency services provided at the hospital or health center and the trauma system that often serves as the network of coordinated trauma care.[5] In all countries, pre-hospital is the predominant place of death occurrence due to injury.[6] Therefore, pre-hospital emergency medical services must be considered in any effort to develop reasonable policies.[7] For strengthening these services, every community should assess its EMS first response system and initiate incremental improvements. An important measure that can be considered in such assessments is response time because EMS are time-driven.[8] This issue is of such importance that, in many jurisdictions, response time standards form the basis of a comprehensive performance measurement system.[9]

Iran, due to its location, is the sixth-most disaster prone country in the world. Moreover, it has been facing many man-made disasters.[10][11][12][13] Therefore, to strengthen the pre-hospital capacity, Ministry of Health and Medical Education (MOHME) has developed triage and evacuation protocols and increased the number of ambulances, EMS stations and air ambulance bases in the last years, especially from 2003 to 2008. Nowadays, Yazd city has 7 stations that provide EMS for trauma patients.[11] We conducted this study to calculate the response time in these stations and compare it with referenced standards and country's approved protocol.

## Materials and Methods

In Iran EMS personnel, legislatively mandated to complete a run report form for each service call they receive. This form includes fields for service (e.g. Service number, date of run), ill or injured demographic information (e.g. Date of birth, sex and town of residence), times and odometer readings (time call received, time that ambulance left the station and time arrived the scene), assessment of patient in scene (e.g. Pulse) and treatments (e.g. Defibrillation). All forms from all stations, monthly submitted to the central station that had responsibility to collect EMS data for further analysis. In the study, we had no sampling and the required data were collected from all 11961 run reports that had been submitted during 2008 and 2009 from all EMS stations in the city. Then we calculated response time for all service calls in minute. We defined response time as the interval starting with the notification of the ambulance unit by dispatch (call time) and ending with the unit's arrival on scene (scene arrival time). All these data were extracted from run report forms. Fortunately, there was not a substantial amount of missing or illogical data for variables that were considered in our analysis. Only 0.02% of reports were missing data on time call received and time that ambulance left the station. We excluded implausibly low and high intervals zero-calculated and greater than 120 minutes response times. For final analysis, we calculated the cumulative percents of calls that have been responded in fractiles (whiten 3, 4, 5, 6, 7 and 8 minutes and more than 8 minutes). Also, we calculated the standard deviation of time response in each fractile using statistical software of Excel.

## Results

**Fig. 1:**
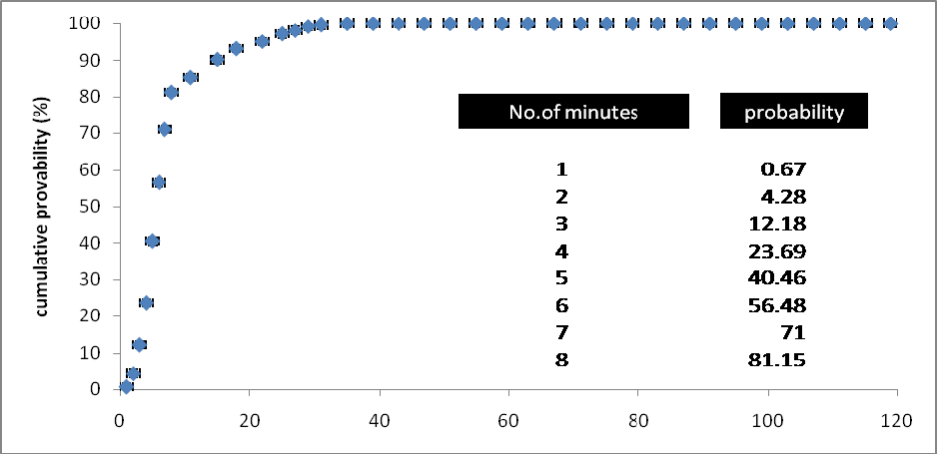
Cumulative probability distribution for response times (min), Yazd EMS: 2008-2009.

Figure 1 displays the cumulative probability distribution for response times. 12.18% of response times were within 3 minutes (SD=0.34 minute), 40.46% within 5 minutes (SD=0.68 minute), 70% within 7 minutes (SD=0.96 minute) and 81.15% of response times were within 8 minutes (SD=1.12 minute), a referenced standard for EMS.

**Fig. 2:**
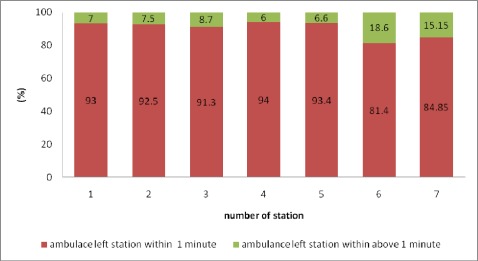
Probability (%) of service calls that ambulance left the station within 1 minute and above after notification.

In this study, we also calculated the time elapsed from notification until time ambulance left station.Figure 2 shows cumulative probability of all service calls that ambulance left the station within 1 minute from notification in each station.

## Discussion 

Iran has been accepted 8 minutes, in 80% of cases as standard response time for EMS. The emergency stations in Yazd city has a good record in comparison to country's approved standard. Also, in the most area of North America, 9 minutes in the 90% of cases was approved as the standard of EMS.[9] The Yazd EMS performance was also properly acceptable in comparison with this standard.

A similar study in Tehran, Iran showed that the average time between notification and ambulance arrival to scene was 12.54 minutes with standard deviation of 1.24 minutes.[14]

Zargar et al. in another study showed that pre hospital average time was 2 hours for trauma patients in Tehran.[6] Results from another study in Western Azerbaijan, Iran showed that the hospital arrival average time was 2 hours and 42 minutes.[2] Comparison of findings in this study with emergency response times in other countries shows more amazing results. For example, one study in Pakistan showed that only 13.6% of patients with acute myocardial infarction symptoms were transmitted to hospital after 1 hour.[15] Also, a review of EMS in Virginia state showed that average time to scene arrival was 12 minutes and 72% of all reported responses were provided less than 10 minutes.[16] A report from Saskatchewan state showed that 88.3% of emergency calls, code 4 (light and Sirens) had been responded within 9 minutes in urban centers, during 2007-2008.[9]

Also, an identical report showed that in Monterrey, Mexico, one unit per 100,000 people managed an average response time of 10 minutes. Hanoi, Vietnam, with one unit for 3 million people, had an average response time of 30 minutes.[17] We must mention that some of the differences between the results of our study and other studies is due to the variations in the ways in which the timeliness of an emergency medical response is measured. The starting point for a response time can range from the time when a call is received at an emergency dispatch center to the time at which a squad has assembled and departs for the scene. Similarly, the way in which the ending time for a response is measured ranges from the time the unit is en route to the time the unit arrives at the patient or the time the unit departs the scene for the hospital.[16]

In this study, response time was evaluated from receiving a call to arrival a unit to scene. So this result can be different from other studies evaluated response time with other methods. But this study had two strengths in comparison with other studies. First we calculated fractile response times. Historically, the mean or average response interval has been used. This is inherently flawed because in roughly 50% of the times, the response interval exceeds the preset standard. A more accurate method involves determining for what percentage of calls the fractile goal is being achieved. The fractile time indicates that a predetermined response interval is being met for a defined percentage of events. This is a much better measure of system performance and thus fractile response intervals should be calculated and used instead of the mean response interval.[8] Also, in this study we had a little missed data. However results of this study showed that the emergency services response time in Yazd, Iran is appropriate in comparison to other countries and standard protocol of country. This can be attributed to many factors such as appropriate development of and investment on EMS in the country in the last years, especially from 2003 to 2008, and also other factors such as geography, service district, distribution of EMS units, population density, traffic and agency staffing levels in the city. Nonetheless, stations that recorded longer response time should analyze distribution process to improve the optimality.

As a conclusion, although the results of this study showed that the pre-hospital response time for trauma injuries, in Yazd city was acceptable, but there are many rooms for improvement. An effective continuum monitoring should be provided for emergency care system by developing data gathering and analyzing capacities, instruments and methods. Also, a national investment for EMS research seems necessary. This investment should help to expand equity in health, not the current inequities.
